# Groundwater rejuvenation in parts of India influenced by water-policy change implementation

**DOI:** 10.1038/s41598-017-07058-2

**Published:** 2017-08-07

**Authors:** Soumendra N. Bhanja, Abhijit Mukherjee, Matthew Rodell, Yoshihide Wada, Siddhartha Chattopadhyay, Isabella Velicogna, Kishore Pangaluru, James S. Famiglietti

**Affiliations:** 1 Hydroscience and Policy Advisory Group, Department of Geology and Geophysics, Indian Institute of Technology, Kharagpur, WB India; 2 Hydrological Sciences Laboratory, NASA Goddard Space Flight Center, Greenbelt, MD USA; 3 School of Environmental Science and Engineering, Indian Institute of Technology, Kharagpur, West Bengal 721302 India; 4 International Institute for Applied Systems Analysis, Laxenburg, Austria; 5 Department of Physical Geography, Utrecht University, Utrecht, The Netherlands; 6 Hydroscience and Policy Advisory Group, Department of Humanities and Social Sciences, Indian Institute of Technology, Kharagpur, WB India; 7 Department of Earth System Science, University of California, Irvine, USA; 8 Jet Propulsion Laboratory, California Institute of Technology, Pasadena, California USA; 9 School of Physical Sciences, University of California, Irvine, USA

## Abstract

The dwindling groundwater resource of India, supporting almost one fifth of the global population and also the largest groundwater user, has been of great concern in recent years. However, in contrary to the well documented Indian groundwater depletion due to rapid and unmanaged groundwater withdrawal, here for the first time, we report regional-scale groundwater storage (GWS) replenishment through long-term (1996–2014, using more than 19000 observation locations) *in situ* and decadal (2003–2014) satellite-based groundwater storage measurements in western and southern parts of India. In parts of western and southern India, *in situ* GWS (GWS_obs_) has been decreasing at the rate of −5.81 ± 0.38 km^3^/year (in 1996–2001) and −0.92 ± 0.12 km^3^/year (in 1996–2002), and reversed to replenish at the rate of 2.04 ± 0.20 km^3^/year (in 2002–2014) and 0.76 ± 0.08 km^3^/year (in 2003–2014), respectively. Here, using statistical analyses and simulation results of groundwater management policy change effect on groundwater storage in western and southern India, we show that paradigm shift in Indian groundwater withdrawal and management policies for sustainable water utilization appear to have started replenishing the aquifers in western and southern parts of India.

## Introduction

**Figure 1:**
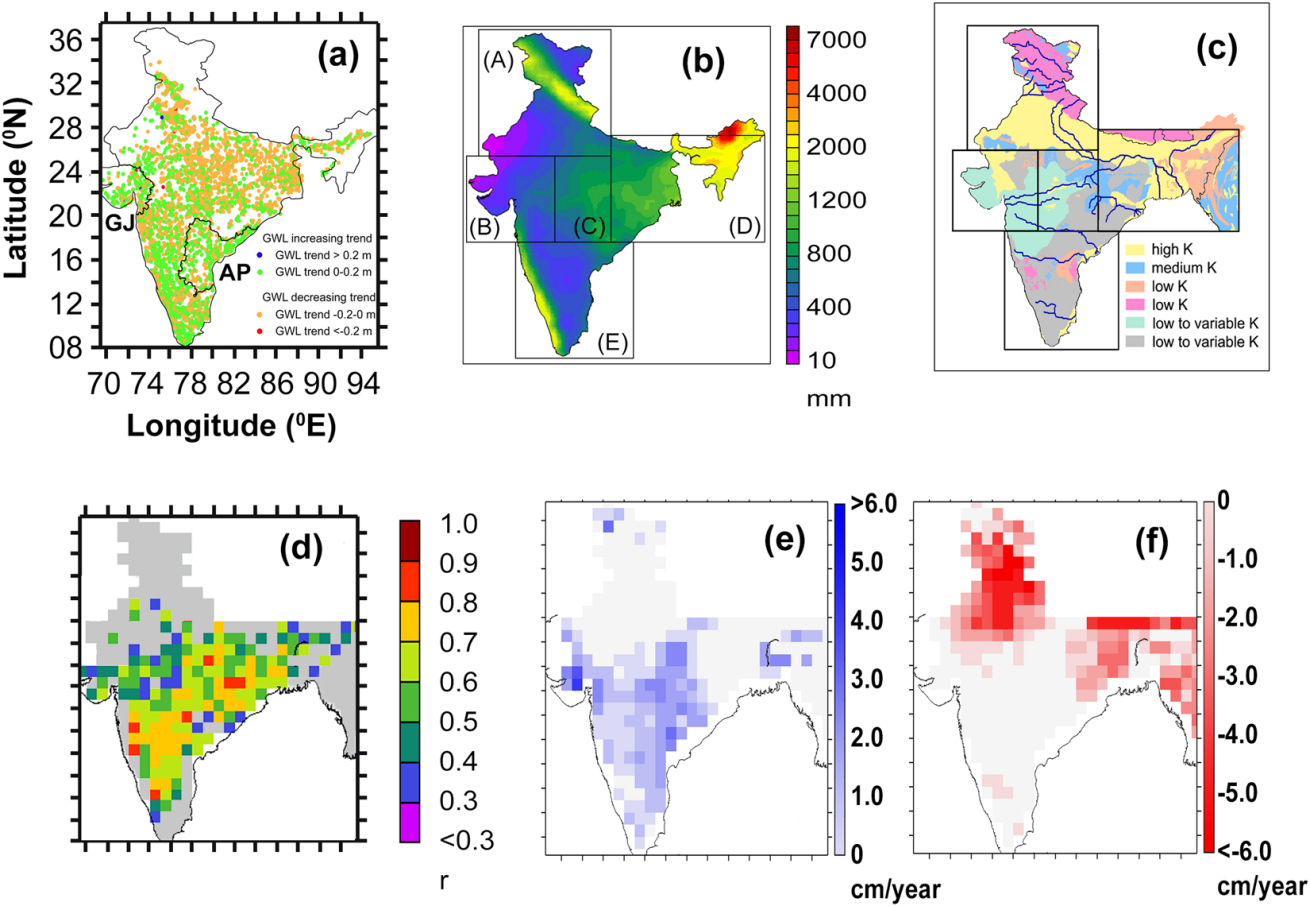
(**a**) The study area including groundwater level (GWL) trend (calculated based on culled, temporally continuous groundwater level measurements [n = 4316] between 1996 and 2014); (**b**) annual mean precipitation (mm/year) between 1979 and 2014. Rectangular outlines indicate the five hydro-meteorological zones (A to E) delineated based on the duration of the hydro-meteorological seasons (monsoon, post-monsoon and pre-monsoon); (**c**) map of different aquifer types, classified based on hydraulic conductivity (K). Major rivers are marked by blue lines; (**d**) map of significant correlation coefficient (r, *p value* 0.05) between GWS_obs_ and GWS_sat_; Maps of trends of (**e**) positive (blue), and (**f**) negative (red) GWS_sat_ anomalies, respectively. All the maps were made using Ferret program (NOAA), QGIS software^47^ and standard graphical illustrators.

Groundwater is the largest liquid freshwater resource of the Earth. It plays a crucial role in human sustenance and global food security by supporting irrigated agriculture^1^. At present, India (Fig. 1a) is undergoing a “groundwater drought”^2^. The country comprises <3% of the terrestrial area and hosts about 19% of the global population. It also covers more than 30% of the global irrigated land^2^ and consumes the largest volume of global groundwater resource (higher than the sum of the total groundwater abstraction of United States and China, the second and third countries, respectively, in the country-wise groundwater utilization list)^3^. The country is witnessing a rapid rise in population, urbanization and change in anthropogenic water use, cropping pattern and lifestyle leading to unsustainable abstraction of available groundwater^4–7^ (e.g. 245 billion cubic meters (BCM) irrigational groundwater abstracted from India alone during 2011 only)^8^, which is at least 25% of the total global groundwater withdrawal^3^. These result to groundwater withdrawal to availability ratio being higher than 0.8 (i.e. more than 80% of the available groundwater has been withdrawn) in most parts of the country^9^. The country has been placed in the top of the list of groundwater depletion (GWD) with 33.9% of the global GWD linked with food production and trade^10^. In recent summers several parts of the country have witnessed law-and-order situation linked with groundwater availability^11^. At least 54% of India has been identified to be highly to extremely water stressed^12^, with present water demand of 712 BCM, projected to be increasing to 833 BCM in 2025 and 899 BCM in 2050^13^. Accordingly, the Indian groundwater scenario has become a global paradigm for future availability and resilience to human strategies. Future groundwater management is challenging with new socio-political alignments at present and near future, and impending, potential climate change^2^.

Causes of groundwater depletion also include inefficient water use practices, wasteful irrigation systems with poor maintenance, and low prices of both power for electricity-driven well pumps and supplied water^14^. Further, the natural groundwater availability and recharge in the region is extremely heterogeneous because of diversity in hydrogeologic set-up^15, 16^ and climatic conditions^15^ (Fig. 1b,c). While, much of the northern India is characterized by the highly groundwater-enriched, porous aquifers of the Indus-Ganges-Brahmaputra (IGB) river basins^15–17^ that are considered as the “bread-basket” of South Asia, much of peninsular India is composed of low-yielding, crystalline aquifers^15, 16^. Similarly, even as the country’s average precipitation is relatively high (~120 cm/year)^18^, it is highly variable both spatially (Asian monsoonal path dependent) and temporally (>75% precipitation during the four monsoon months)^18^, resulting to the Indian climate varying from extremely arid to some of the wettest places on Earth (Fig. 1b). Thus, the entire study area has to be classified into five different Hydro-meteorological zones (HMZ) with similar climatic pattern (see methods and supplementary information).

However, contrary to the aforesaid condition of groundwater depletion, general observations suggest groundwater replenishment trends in some parts of India, with groundwater storage (GWS) renewal trends of up to ~4 m rise in groundwater level in recent years^19^, specifically in parts of the western Indian state of Gujarat (HMZ B), and south Indian state of Andhra Pradesh (HMZ E, undivided state [including present Telengana state] indicated in Fig. 1a). While, unregulated abstraction for enhanced irrigation of water-intensive cultivation (e.g. boro rice, including Basmati)^19^ is resulting to one of the most rapid and drastic groundwater depletion in human history^3^, recent paradigm shift in Indian central/state government management strategies on groundwater withdrawal and stress (e.g. Pradhan Mantri Krishi Sinchayee Yojana) will likely start to demonstrate its results in near future. Policies like restriction of subsidized electricity for irrigation^20^, separate electricity distribution for agricultural purpose (Jyotigram Yojana)^21^, construction of large-scale, regional enhanced recharge systems in water-stressed crystalline aquifers (e.g. ~700 million USD allocated to Tapti river mega recharge project^22^), artificial recharge of 85 BCM/year in ~0.5 million km^2^ through ~10 million structures^23^, enhanced recharge by interlinking of river catchments (e.g. Narmada-Sabarmati interlinking^24^), will probably start replenishing the aquifers^25^ by increasing groundwater storage in near future. To quantify the replenishment trends, we used two-decade (1996–2014) long *in situ* and decadal (2003–2014) satellite-based measurements of groundwater conditions. We also used robust statistical approaches and a global-scale hydrological model simulation results in order to show the influence of water management policy change (based on data availability) in replenishment of groundwater storage in western and southern parts of the country.

## Result and Discussion

### Groundwater storage estimates

**Figure 2:**
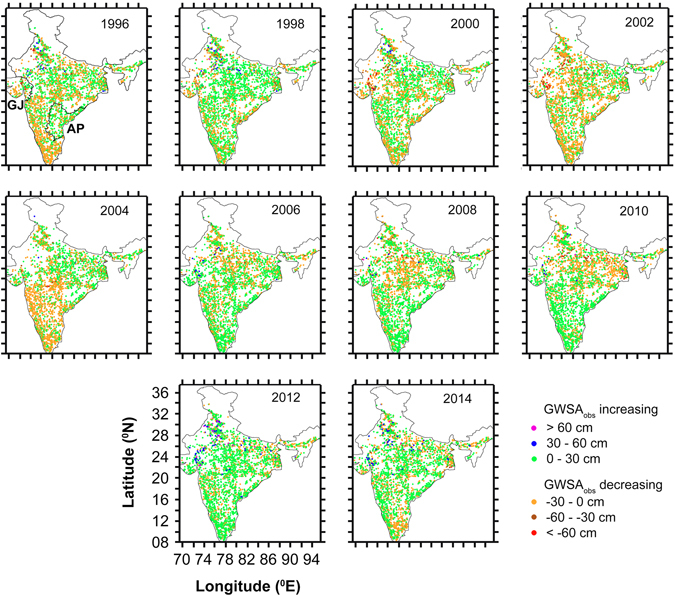
Maps of annual *in situ* groundwater storage anomalies (GWSA_obs_) over the study area between 1996 and 2014. GJ (Gujarat) and AP (Andhra Pradesh) indicate the location of specific study areas. All of the maps were made using QGIS software^47^.

We used seasonal *in situ* groundwater storage anomalies (GWSA_obs_) and monthly groundwater storage anomalies (GWSA_sat_, 2003–2014), calculated using the Gravity Recovery and Climate Experiment (GRACE) satellite mission and land surface model-simulated soil moisture and surface water equivalents, to quantify the groundwater storage anomaly trends in the Indian region (Fig. S2a–l). The two anomalies demonstrate good correlation with each other (*p value* < 0.05) (Fig. 1d), and were found to substantiate each other for the overlapped time period (detailed information on validation of satellite-based products in the study region can be found in Bhanja *et al*.^16^). Trend analyses of groundwater level anomalies (GWLA), GWSA_obs_ (1996–2014) and GWSA_sat_ (2003–2014) indicate increasing trends in western (HMZ B) and southern (HMZ E) India, respectively (Figs 1a,e and 2), corroborating well with the aforesaid general observations of groundwater rejuvenation. GWSA_obs_ data indicate renewal of GWS in HMZs B and E at a rate of 1.06 ± 0.03, and 0.31 ± 0.02 km^3^/year, on the other hand, the HMZs A and D have been subjected to rapid GWS depletion at a rate of 4.55 ± 0.11 km^3^/year and 3.59 ± 0.14 km^3^/year, respectively (Fig. 2). Satellite-based observation shows, the western (HMZ B) and southern (HMZ E) India, are experiencing GWSA_sat_ renewal at a rate of 0.53 ± 0.30 and 0.69 ± 0.27 cm/year (2.29 ± 1.32 and 3.90 ± 1.50 km^3^/year, with *p value* < 0.05), respectively (Fig. S2a–l). Concurrently, rapid groundwater depletion (Fig. 1f) was observed in northern (HMZ A) and eastern (HMZ E) India, respectively, at a rate of 1.40 ± 0.14 and 1.16 ± 0.35 cm/year (14.02 ± 1.37 and 14.49 ± 4.36 km^3^/year, *p* < 0.05). These observations are in agreement with previous *in situ* observations over IGB basin (depletion at a rate of ~8 km^3^/year between 2000 and 2012)^17^, and satellite-based observations of GWS depletion from the general areas, e.g. northwest India (17.7 km^3^/year, 2002–08)^4^ and northern (54 km^3^/year, 2002–08)^5^ India, Ganges basin (1.25 cm/year, 2003–14)^7^ and Bangladesh (located within HMZ D)^6^.

### Groundwater storage rejuvenation in specific study areas

**Figure 3:**
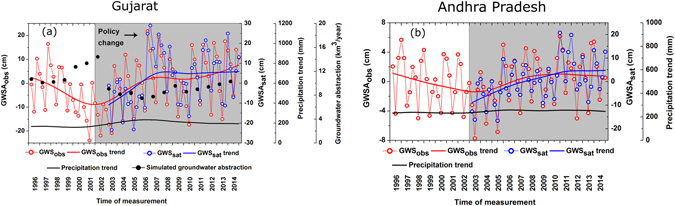
Annual GWSA_obs_, GWSA_sat_, and their Hodrick-Prescott (HP) trends for (**a**) Gujarat (in HMZ B) and (**b**) Andhra Pradesh (in HMZ E). The grey area represents the time-period for implementation of groundwater management policy change. Change in simulated groundwater abstraction from PCR-GLOBWB simulation for Gujarat is shown in (**a**). Precipitation HP trends are shown using black line.

In order to find the potential effects of groundwater management policies, long term, temporally continuous, groundwater level data (1996–2014) from specific study areas of western India (Indian state Gujarat, n = 177 *in situ* locations) and southern India (Indian state Andhra Pradesh, n = 350 *in situ* locations) were studied in details. The GWSA_obs_ were decomposed between trends and cycles using non-parametric Hodrick-Prescott (HP)^26^ filter approach. The HP filter analyses show declining groundwater trends from (pre-)1996 time, with GWSA_obs_ decreasing at the rate of 5.81 ± 0.38 km^3^/year (linear trend), and reversal to increasing trends (GWSA_obs_ increasing at the rate of 2.04 ± 0.20 km^3^/year) from 2002 in Gujarat (Figs 3a and S5). Similarly, declining trend (0.92 ± 0.12 km^3^/year) of Andhra Pradesh from (pre-)1996 reversed around 2003, increasing at the rate of 0.76 ± 0.08 km^3^/year, respectively (Figs 3a and S6). The trend reversals are found to be synchronous with various groundwater policy changes in these two states. For the same time period (i.e. 1996–2014), there were no visible changes in HP trends of precipitation for the two study areas (Fig. 3a and b), thus reducing possibilities of much influence of rainfall on the aforesaid trends of groundwater storage increase or decrease.

### Influences of water management policy change on groundwater storage

Reduction in agricultural power supply from 16 billion units to 10 billion units between 2001 and 2006^27^ might results in reduction in irrigational groundwater quantities (as electricity-driven wells are responsible for over 90% of the groundwater withdrawal)^21^. The groundwater withdrawal is assumed to be directly proportional to the agricultural electricity usage that is required to operate the pump (see methods). We tried to understand the potential effects of these policy changes (reduction of agricultural electricity usage, AEU) on groundwater storage anomaly in Gujarat between 1996 and 2012 (based on data availability) by using first-order Bayesian Vector Autoregression (BVAR). We have used Minnesota prior for our estimation, which automatically takes care of the potential non-stationarity of the data. Based on the BVAR estimate, we have also generated Impulse Response Functions (IRFs) to uncover the dynamics of AEU and GWSA_obs_. We have used Cholesky Decomposition for one standard deviation shock to innovation and ordering AEU to GWS to generate the IRFs (Fig. S4). Results show electricity usage for groundwater irrigation was found to have significant (*p value* < 0.05) negative impact on GWSA_obs_ in Gujarat, implying that reduction of electricity usage leads to increase in GWSA_obs_. We have also performed the Granger causality analyses^28–30^, in order to study the causal relationship between reduction in electricity usage and GWSA_obs_. Result indicates AEU reduction Granger causes GWSA_obs_ in Gujarat. In order to show the effects of policy change on GWS in Gujarat, we have also used a global-scale hydrological model, PCR-GLOBWB^31, 32^, based on available policy change information. The model was operated in a Baseline Scenario (BS) using observed meteorologic and other model forcing parameters (i.e. precipitation, irrigation data, domestic and industrial water use etc.; *see methods and supplementary information*). An Hypothetical Test Scenario (HTS) has been considered, which includes BS scenario along with updated groundwater policy change information. We used the electricity reduction information in HTS simulation of Gujarat (i.e. lowering ~1/3 of agricultural power consumption) and compared the output GWS with that of the BS. The simulation results support our observation with an estimated increase in GWS by 3.2 to 4.4 km^3^/year in Gujarat *(p* < 0.05*)* during 2003–2014 on comparing the GWS for 1996–2002. It is interesting to note that the total precipitation rate in the area has not increased, even decrease in mean annual precipitation has been observed (linearly decreasing at a rate of 9.11 ± 4.45 mm/year) during 2002–2014.

Similarly, in Andhra Pradesh, UN FAO programs between 2004 and 2008, trained farmers on groundwater sustainable management practises^33^. Prior to the UN FAO program, another project named APWELL^34^ was linked with groundwater development in Andhra Pradesh. Substantial increase in surface water irrigation (SWI) have been observed 1996 onwards (>18% increase in SWI between 2003–2011 from 2002 level)^35^, thereby possibly inducing enhanced non-meteoric recharge^36^. The IRFs has been studied using first-order BVAR using Minnesota prior. We have used Cholesky Decomposition for one standard deviation shock to innovation and ordering SWI to GWSA_obs_ for 1 standard deviation innovations shock (Fig. S5) between 1996 and 2011 (based on data availability). Results show significant (*p value* < 0.01) positive impact of SWI on GWSA_obs_ (Table S2, Fig. S5). It is also found that SWI Granger causes GWSA_obs_, thus supporting our hypothesis of groundwater rejuvenation in AP from 2004 onward, being possibly associated with enhanced SWI.

### Assumptions and limitations

We inferred the increasing trends of groundwater to be related to groundwater management strategy adaptation in the detailed study areas (e.g. decreasing power subsidy or increasing artificial recharge by creating surface water bodies), through advanced statistical approaches and simulation of a global-scale hydrological model. In present knowledge scenario, impacts of some of the water management issues are almost impossible to quantify, for example, the effect of change in farmer management practice on groundwater resources in Andhra Pradesh etc. Total groundwater abstraction (Q) for Gujarat can be computed following Eq. 1 (see methods). It can be said that, groundwater abstraction is directly proportional to the electricity consumption. The information has been incorporated in PCR-GLOBWB to simulate change in groundwater storage in Gujarat. The policy related information are available from 1996 to 2012 (Gujarat) and 2011 (Andhra Pradesh), hence, the analyses are conducted up to the time period of data availability. The PCR-GLOBWB simulations are extended for two more years i.e. 2013 and 2014 using the values of 2012.

## Conclusions

We conclude that in India, where huge groundwater consumption is widely known to be leading to severe dwindling of groundwater resource in recent times, previously unreported, discernable GWS replenishment can also be observed in certain Indian regions. Specifically, in parts of the western (HMZ B) and southern (HMZ E) India, GWS_obs_ decreased at the rate of −5.81 ± 0.38 km^3^/year (in 1996–2001) and −0.92 ± 0.12 km^3^/year (in 1996–2002), and reversed to replenish at the rate of 2.04 ± 0.20 km^3^/year (in 2002–2014) and 0.76 ± 0.08 km^3^/year (in 2003–2014), respectively. This groundwater storage rejuvenation may possibly be attributed to implementation of ingenious groundwater management strategies in both Indian public and private sectors. We have tested and substantiated this hypothesis in the two areas in western and southern India, by Bayesian VAR estimates, causality test and simulating the effects of water management policy changes, as applicable, by using a global hydrological model. Thus, while the northern and eastern parts of India are still undergoing acute usable groundwater depletion and stress, encouraging, replenishing groundwater scenarios are detected in the western and southern India under proper water resource management practices.

## Methods

### Study area

India has extremely heterogeneous spatial climatic pattern with variable precipitation and other meteoric water component (e.g. humidity). To overcome this meteorological heterogeneity, for this study, the region was divided into 5 hydro-meteorological zones (HMZ) on the basis of their precipitation and specific humidity pattern. We used long term monthly mean precipitation data set [Global Historical Climatological Network (GHCN)] from the year 1960 to 2010 for >120 locations spread over the study area. We filtered on availability of at least 70% of continuous data. As a result, precipitation data over 37 locations were used in the present study. We used European Center for Medium Range Weather Forecasting (ECMWF) reanalysis^37^ (ERA Interim) simulation of specific humidity (SH) in the present study area between 1979 and 2012 to constrain the boundary of the hydro-meteorological seasons (Supplementary Information) corresponding to different hydro-meteorologic zones (HMZs) (Supplementary Information). We used monthly meteorological sub-division-wise *in situ* precipitation data for Gujarat and Andhra Pradesh between 1996 and 2014^38^. We used 1^0^ × 1^0^ monthly gridded precipitation data from ECMWF’s simulation of reanalyzed (ERA Interim)^37^ total precipitation product from 2003 to 2014 to prepare the Fig. 1b.

### *In situ* groundwater level measurements

We have collected groundwater level (GWL) data from Central Ground Water Board (CGWB, India) repository for measurements between January 1996 and November 2014 from mostly unconfined aquifers (~87%)^39^ for a total number of observation wells in 19278 locations, having GWL data collection of maximum four times a year (late post-monsoon [January], pre-monsoon [May], monsoon [August] and early post-monsoon [November]). In order to use the continuous data for anomaly analyses, we have selected the locations with at least three seasonal data in every year and outliers were removed following Tukey’s fence approach^40^, reduced the usable well numbers to 4316. Linear trend in water level data were calculated using a linear regression model^17^. GWL anomaly (GWLA) for each of these wells was computed after subtraction of all time mean depth to water table from absolute depth of water table in each season and the sign made reversed for depth below the surface convention. The GWLA information has been transformed to GWS anomalies (GWSA_obs_) after incorporating specific yield information for each well^16, 18^ (details can be found within Supplementary information). The median values of GWSA_obs_ within a grid cell were used to make 1^0^ × 1^0^ gridded data for the whole study area and compared with GWSA_sat_.

### Satellite-based groundwater storage estimation

We used 133 monthly GRACE RL05M mascon solutions, obtained from the Jet Propulsion Laboratory, National Aeronautics and Space Administration (NASA)^16, 41^ between January 2003 and December 2014, to determine terrestrial water storage (TWS) anomaly. Anomalies were calculated related to the mean during the entire period. In order to determine groundwater storage (GWS) anomalies, anomalies of other water components of terrestrial water cycle i.e. soil moisture (SM), and surface water (SW) equivalents were removed from TWS anomalies. We used the Global Land Data Assimilation System (GLDAS)^42^ for SM and SW estimates in the corresponding 133 GRACE measurement months over the study period. We used combination of three different models’ output, Community Land Model (CLM2), Variable Infiltration Capacity (VIC), and NOAH to remove any bias associated with a single model. Before comparing with GWLA and GWSA, we used GRACE based estimates corresponding to the observational time period only i.e. four times a year between 2005 and 2013. Subsequently, we removed 36-monthly mean from TWS, SM, and SR of a particular month and followed the same procedure discussed above for calculating GWS_sat_ anomaly.

### Quantification of influence of policy change on groundwater

The non-parametric trend analysis approach, the Hodrick-Prescott (HP) statistical filter^26^ is a robust technique used to decompose time series data into trend and cycle (see SI for more details). It allows data to reveal its own trend and does not impose any trend structure on the data arbitrarily. As a result it can even capture nonlinearity in trend if it is present in the data. HP filter was used in trend analysis of GWL in the high resolution study area (Gujarat and Andhra Pradesh). We have also used first order Bayesian vector auto-regression (VAR) analysis^43^ and test for Granger causality^28^ for GWL and GWI in GJ and SWI in AP, in order to show causal relationships. A best practice parameter (BP) was also introduced in AP to account for the farmers training on sustainable groundwater management practice.

Total groundwater abstraction (Q) for Gujarat can be computed following the equation^36^:

1 $$Q=\frac{N\times r\times t}{A}$$

where, N = total number of pumps, r = pumpage rate, t = time and A = geographical area. Assuming *N* and *r* are fixed over time, reduction in electricity consumption would actually lead to decrease in *t*.

### Error analysis

We have estimated one-sigma trend error in the GRACE TWS anomaly estimates (σ_TWS_). Errors associated with soil moisture anomaly (σ_SM_) and surface water anomalies (σ_SR_) were determined by estimating the standard deviations of the trends of the three GLDAS models. Subsequently, error estimates in the trend of GWS anomaly (σ_GWS_) are estimated following the equation,

2 $${\sigma }_{{\rm{GWS}}}=\surd [{({\sigma }_{{\rm{TWS}}})}^{2}+{({\sigma }_{{\rm{SM}}})}^{2}+{({\sigma }_{{\rm{SR}}})}^{2}]$$

Errors in GWS_obs_ and precipitation data were obtained by estimating one-sigma trend error for calculating the linear trend.

### Hydrological model simulation

We have simulated the effects of policy change using a widely used, global-scale hydrological model, PCR-GLOBWB, that simulates surface water and groundwater resources at 0.1^0^ × 0.1^0^ global grids (i.e., roughly 10 km by 10 km at the equator) on a daily time step^31, 32^. The model simulates water fluxes (e.g., runoff, infiltration, percolation) and water storage (e.g., soil moisture, groundwater), and allows water exchange in two vertically stacked soil layer and an underlying groundwater layer, which is the part of deeper soil layers that is not directly linked with vegetation. The groundwater layer was directly fed by active recharge through upper soil layers. Groundwater recharge was balanced by capillary rise if the water table lies within 5 m below the ground surface^31, 44^. Aquifers were parameterized based on lithology and topography of the region and represented as linear reservoir model^45^. Observation based WATCH ERA-Interim (WFDEI) was used in PCR-GLOBWB for climate forcing^46^, the observed precipitation data has been used here. More information on model description can be found in supplementary information.

## Electronic supplementary material

Supplementary Information
