# Supplementary material for: Groundwater rejuvenation in parts of India influenced by water-policy change implementation
Source: Sci Rep. 2017 Aug 7;7:7453. doi: 10.1038/s41598-017-07058-2 (PMC5547062; doi:10.1038/s41598-017-07058-2)
Supplement: Supplementary file 1 — Supplementary Information [file 41598_2017_7058_MOESM1_ESM.pdf]

Groundwater rejuvenation in parts of India influenced by water-policy change implementation

Soumendra N. Bhanja[1,2], Abhijit Mukherjee[1,3*], Matthew Rodell[2], Yoshihide Wada[4,5,6,7], Siddhartha Chattopadhyay[8], Isabella Velicogna[9,10], Kishore Pangaluru[9] and James S. Famiglietti[10,11]

[1]Department of Geology and Geophysics, Indian Institute of Technology Kharagpur, WB, India

[2]Hydrological Sciences Laboratory, NASA Goddard Space Flight Center, Greenbelt, MD, USA

[3]School of Environmental Science and Engineering, Indian Institute of Technology Kharagpur, West Bengal 721302, India

[4]International Institute for Applied Systems Analysis, Laxenburg, Austria

[5]Department of Physical Geography, Utrecht University, Utrecht, The Netherlands

[6]NASA Goddard Institute for Space Studies, New York, NY10025, United States

[7]Center for Climate Systems Research, Columbia University, New York, NY, United States

[8]Department of Humanities and Social Sciences, Indian Institute of Technology Kharagpur, WB, India

[9]Department of Earth System Science, University of California-Irvine, USA

[10]Jet Propulsion Laboratory, California Institute of Technology, Pasadena, California, USA

[11]School of Physical Sciences, University of California-Irvine, USA

Contents of Supplementary Information

S1 Hydrometeorologic zone (HMZ) delineation

S2 GRACE mascon solution

S3 Satellite-based estimates using combination of 3 land surface models and using individual ones

S4 PCR-GLOBWB simulation for detailed study area

S5 Statistical analyses used

S6 Specific yield information

We used long term monthly mean precipitation data sets [Global Historical Climatological Network (GHCN)] from the year 1960 to 2010 for more than 120 locations spread over the study area. Continuous data were not available for several locations and hence the data were screened and restricted the selection of a location on the basis of availability of at least 70% of continuous

time series data. Accordingly, we selected 37 locations that are evenly distributed over the entire study area. We used European Center for Medium Range Weather Forecasting (ECMWF) reanalysis[30] (ERA Interim) simulation output of specific humidity (SH) over the selected locations between 1979 and 2012 to constrain the boundary of the hydro-meteorological seasons. Precipitation as well as SH data exhibited higher values during monsoon and distinguishing the boundary between monsoon and non-monsoonal seasons. Pre-monsoon and post-monsoon seasons were defined by determining the rate of change in SH data, as precipitation is negligible during these seasons for most of the locations. The SH values showed decrease in trend immediately after monsoon, and then increasing trend from the advent of the monsoon. This sudden shift (decreasing to increasing) in SH values marked the boundary between post-monsoon and pre-monsoon in the present study. After defining hydrological seasons, we imposed inter-quartile range cut off for selecting the months that composed the seasons and accordingly, defined five hydro-meteorologic zones (HMZs). For example, location-wise long-term monthly mean of precipitation and SH values are shown in Supplementary Figure 1. Each two locations represent data from a HMZ in the following order, northern, western, central, eastern and southern. The seasons for the hydrologic year were defined for each of the HMZs as: Northern region [monsoon: June-September; post-monsoon: October-January; pre-monsoon: February-May]; Western region [monsoon: June-September; post-monsoon: October-February; pre-monsoon: March-May]; Central region [monsoon: June-September; post-monsoon: October-March; pre-monsoon: April-May]; Eastern region [monsoon: June-September; post-monsoon: October-January; pre-monsoon: February-May]; Southern region [monsoon: June-October; post-monsoon: November-January; pre-monsoon: February-May].

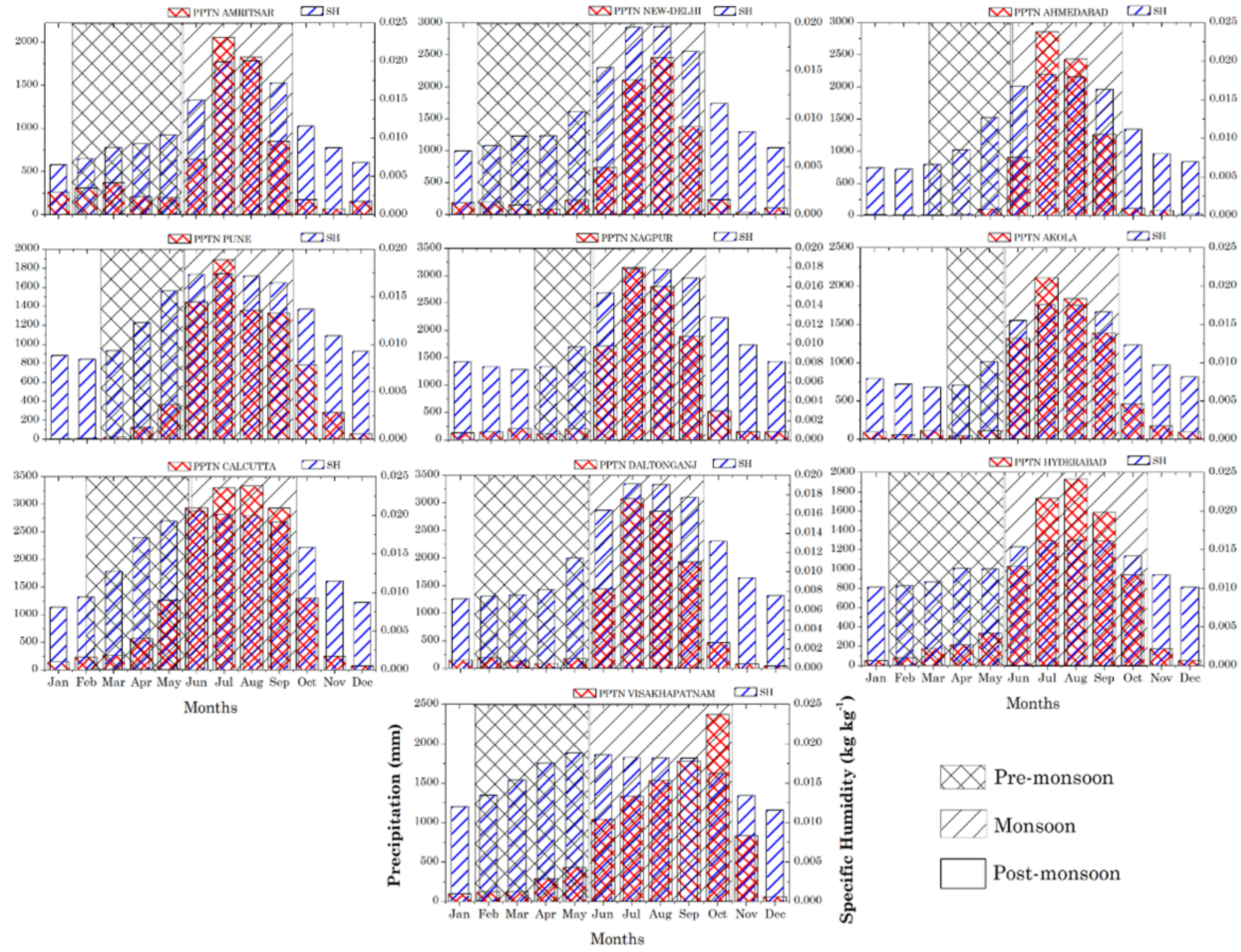

Fig. S1: Long-term mean precipitation (1960 to 2010) and specific humidity (1979 to 2012) mean values over 10 different locations indicating hydrometeorologic seasons

S2 GRACE mascon solution

GRACE JPL MASCON solutions (RL05M) are used here between January 2003 and December 2014 in order to determine terrestrial water storage (TWS) (Watkins et al., 2015). Monthly gravity field variations were parameterized using 4,551 equal-area 3^0 surface spherical cap mascons. Inter-satellite range rate measurements between the two GRACE satellites are used for estimating variations in gravity field through application of partial derivatives. Degree 2 and order 0 coefficients are replaced in JPL processed level 1 GRACE observations by the coefficients derived from Satellite Laser Ranging reported by Cheng et al. (2011). Degree 1 coefficients are derived from a process described elsewhere (Swenson et al., 2008). Glacial isostatic adjustment (GIA) in the data has been removed following the process developed by Geruo and Wahr (2013). In order to minimize the measurement errors, a-priori constraints have been applied in gravity fields of equal-area spherical mascons.

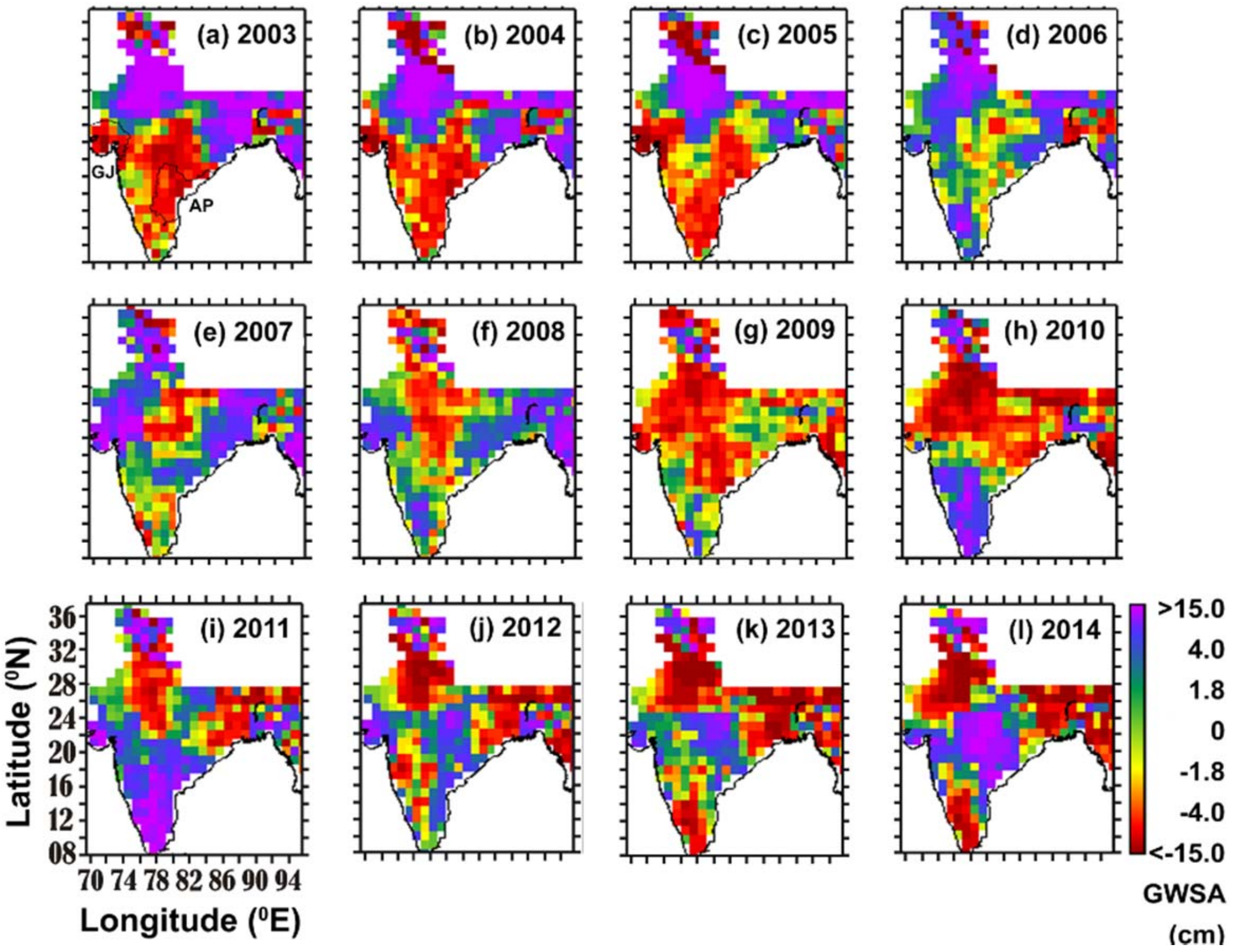

Fig. S2 (a) to (l): Maps of annual groundwater storage anomalies ($GWSA_{sat}$) over the study area. All the maps were made using Ferret program (NOAA)

S3 Satellite-based estimates using combination of 3 land surface models and using individual ones

We have performed the estimation of satellite-based GWS using a combination of 3 land surface models (LSMs) and also using estimates from individual models between 2005 and 2013, details can be found in Bhanja et al. (2016). The RMSE and Pearson's correlation coefficient are shown for the GWS obtained using 3 LSMs' combination and using each of the LSMs with the GWS obtained from in situ observation wells on 12 largest river basins in India (Bhanja et al., 2016). On the basis of selection of an individual model's estimate, the satellite-based GWS showing good match with in situ GWS in some of the basins, however, none of the models was showing consistent result while comparing the estimates in all of the river basins. In general, the combination of the models provides best satellite-based estimates (details can be found in Bhanja et al., 2016).

Table S1: RMSE values of in situ and satellite-based GWS anomaly calculated using a combination of 3 LSMs and using individual LSMs' output. Best matches are written in bold font

Basin code	Basin name	GRACE-based GWS			
		Combination	CLM	NOAH	VIC
1	Indus Basin (Indian part)	3.24	**2.94**	3.16	4.40
2a	Ganges Basin (Indian part)	**5.72**	7.12	6.29	6.48
2b	Brahmaputra Basin (Indian part)	9.45	9.76	**6.20**	7.23
3	Godavari Basin	6.29	10.63	**6.25**	8.29
4	Krishna Basin	**8.59**	17.93	14.13	14.85
5	Cauvery Basin	15.17	12.59	**10.02**	11.37
8	Mahanadi Basin	**6.20**	9.55	7.44	7.32
12	Narmada Basin	8.35	9.85	**6.73**	8.52
13	Tapi Basin	**7.93**	12.43	9.62	23.99
14	West flowing rivers South of Tapi Basin	13.90	**8.06**	8.77	11.47
18	East flowing rivers between Pennar and Cauvery Basin	8.92	**8.56**	9.11	9.61
20	West flowing rivers of Kutch and Saurashtra including Luni Basin	**7.52**	9.55	12.69	11.96

Table S2: Pearson's correlation analysis of in situ and satellite-based GWS anomaly calculated using a combination of 3 LSMs and using individual LSMs' output. Best matches are written in bold font

Basin code	Basin name	GRACE-based GWS			
		Combination	CLM	NOAH	VIC

1	Indus Basin (Indian part)	0.71	**0.84**	0.71	0.47
2a	Ganges Basin (Indian part)	0.83	**0.85**	0.65	0.81
2b	Brahmaputra Basin (Indian part)	0.47	0.57	**0.69**	0.68
3	Godavari Basin	**0.89**	0.82	0.87	0.84
4	Krishna Basin	**0.91**	0.81	0.77	0.72
5	Cauvery Basin	**0.75**	0.34	-0.03	0.25
8	Mahanadi Basin	**0.83**	0.66	0.55	0.74
12	Narmada Basin	0.56	0.67	**0.69**	0.65
13	Tapi Basin	**0.77**	0.49	-0.24	-0.35
14	West flowing rivers South of Tapi Basin	-0.33	0.49	0.53	**0.55**
18	East flowing rivers between Pennar and Cauvery Basin	0.33	**0.50**	0.31	0.34
20	West flowing rivers of Kutch and Saurashtra including Luni Basin	**0.70**	0.36	-0.04	0.03

S4 PCR-GLOBWB simulation for detailed study area

In PCR-GLOBWB, total groundwater recharge (R_{sim}) is a combination of diffuse recharge through precipitation, and irrigational return flow; recharge was estimated by subtracting capillary rise from deep percolation [*Wada et al.,* 2014]. The capillary rise is calculated by considering moisture flux in an upward direction under an existing upward gradient condition with soil moisture concentration lying below field capacity, and the amount could not exceed groundwater storage of underlying groundwater layer [*Wada et al.*, 2014]. In order to produce a more realistic soil moisture, evaporation and transpiration, the irrigation scheme separately parameterizes paddy and non-paddy crops. The scheme also dynamically balance daily amount of surface and soil water by considering feedback from irrigation water. In the paddy fields, a 50 mm surface water depth [*Wisser et al.,* 2010] was considered up to a late crop-growing stage and no irrigation phase was considered (~20 days) before the harvesting period [*Wada et al.,* 2014].

Irrigated area was estimated from country-specific information provided by FAOSTAT (http://faostat3.fao.org/home/E). Domestic and industrial water requirements were also

calculated on a daily basis. Domestic and livestock water requirement data in 2000 were collected from FAOSTAT and the daily water requirement was calculated as a function of temperature [*Wada et al.,* 2011]. Change in water use intensity as a factor of economic and technological development also considered here [*Wada et al.,* 2014]. Simulated Topological Networks [*Vörösmarty et al.,* 2000] data were employed to simulate direct runoff, interflow and base flow. Irrigation water allocation was calculated from available surface water resources and readily available groundwater.

Tall and short vegetation, paddy rice, non-paddy crops, rain-fed crop, open water, different soil types are considered following FAO Digital Soil Map of the World (FAO, 2003) in each of the grid cell. The improved ARNO scheme are used to estimate saturated soil area fractions (Todini, 1996; Hagemann and Gates, 2003) and the groundwater depth frequency distribution are obtained from the HYDRO1k Elevation Derivative Database (HYDRO1k; U.S. Geological Survey Center for Earth Resources Observation and Science; https://lta.cr.usgs.gov/HYDRO1K/). The Hypothetical Test Scenario (HTS) considered in this study includes model simulation using all of the above mentioned data along with policy change related parameters in Gujarat. Global water use simulations were done using dynamic meteorological forcing parameters, i.e. temperature and precipitation data from ERA Interim and MERRA reanalysis products and reference evapotranspiration was calculated following FAO guidelines (with Penman-Monteith equation) in a grass surface [*Wada et al.,* 2014].

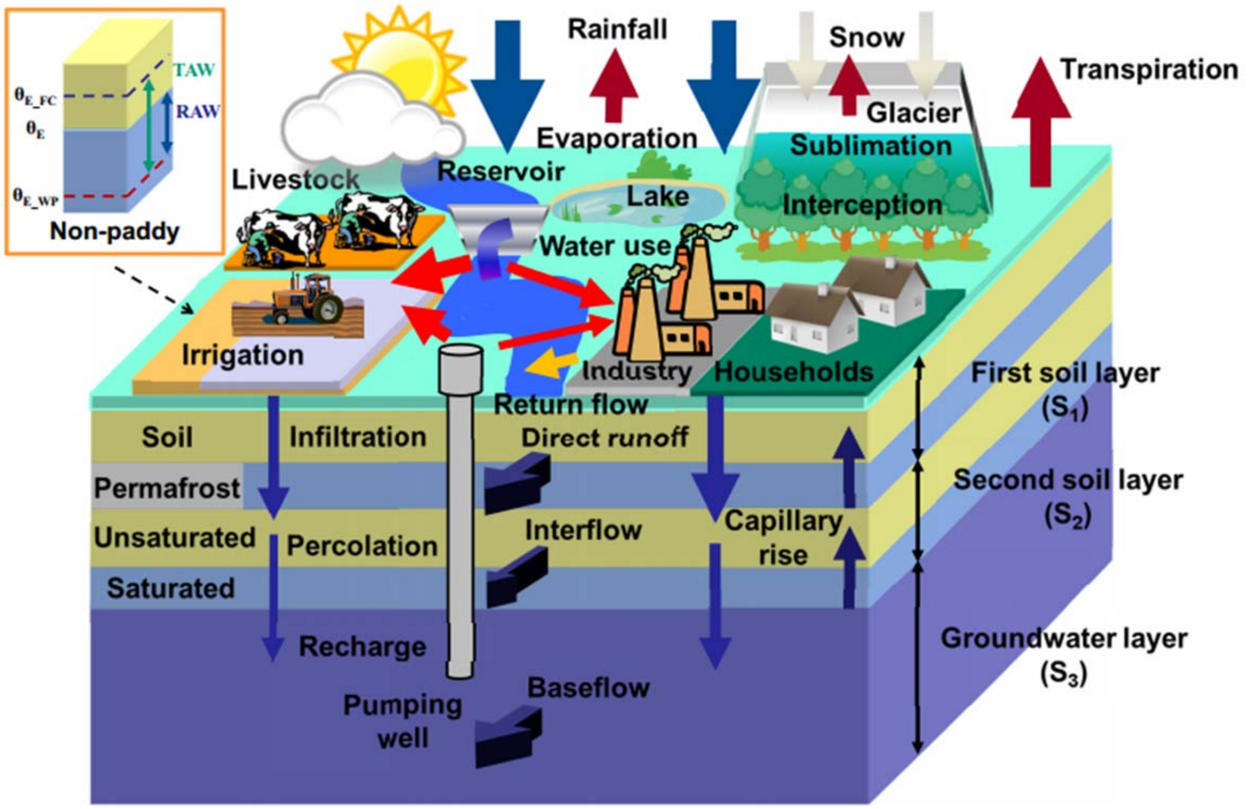

Fig. S3: Schematic diagram of PCR-GLOBWB operation

S5 Statistical analyses used

Hodrick-Prescott (HP) filter analysis:

We have tried to estimate trend using linear regression, although non-linearity in the data restricts us to rely totally in linear trend analysis. We used Hodrick-Prescott (HP) filter [Hodrick and Prescott, 1997], a non-parametric, non-linear trend analysis widely used over the years. The HP filter separates trend (T_t) and cyclical (c_t) components from the data time series (y_t).

$$y_t = T_t + c_t \quad (1)$$

In order to separate the trend component, the HP filter solves the following equation:

$$\text{Min (T)} \sum_{t=1}^{T}((y_t - T_t)^2 + \lambda((T_{t+1} - T_t) - (T_t - T_{t-1}))^2 \quad (2)$$

The smoothing parameter (λ) is a positive number, which reduce the variability in the cyclical component [Hodrick and Prescott, 1997]. We have chosen the value of λ for the quarterly data, 1600 [Hodrick and Prescott, 1997; Ravn and Uhlig, 2002]. Detailed descriptions on HP filter can be found in Hodrick and Prescott [1997] and Ravn and Uhlig [2002].

Bayesian VAR model analyses:

We have estimated Bayesian VAR and tested Granger causality for Gujarat and Andhra Pradesh. Lag values of surface water irrigation (SWI) has a significant (p value <0.05) positive impact on GWS anomaly in Andhra Pradesh. Lag values of groundwater irrigation (GWI) has a significant (*p value* <0.05) negative impact on GWS anomaly in Gujarat- this implies GWI Granger causes GWS anomaly in Gujarat, as a result, GWS increases after 2002-2003 due to reduction in electricity usage for groundwater pumping.

Table S1: Bayesian VAR parameters [t-statistic values] for Gujarat

	GWS_t
GWS_{t-1}	0.10<br><br>[1.34]
GWI_{t-1}	-0.00<br><br>[-2.39]*

*statistically significant at 5% confidence level

Table S2: Bayesian VAR parameters [t-statistic values] for Andhra Pradesh

	GWS_t
GWS_{t-1}	0.06<br><br>[0.76]
SWI_{t-1}	0.27<br><br>[2.57]*
BP_{t-1}	1.29<br><br>[2.19]*

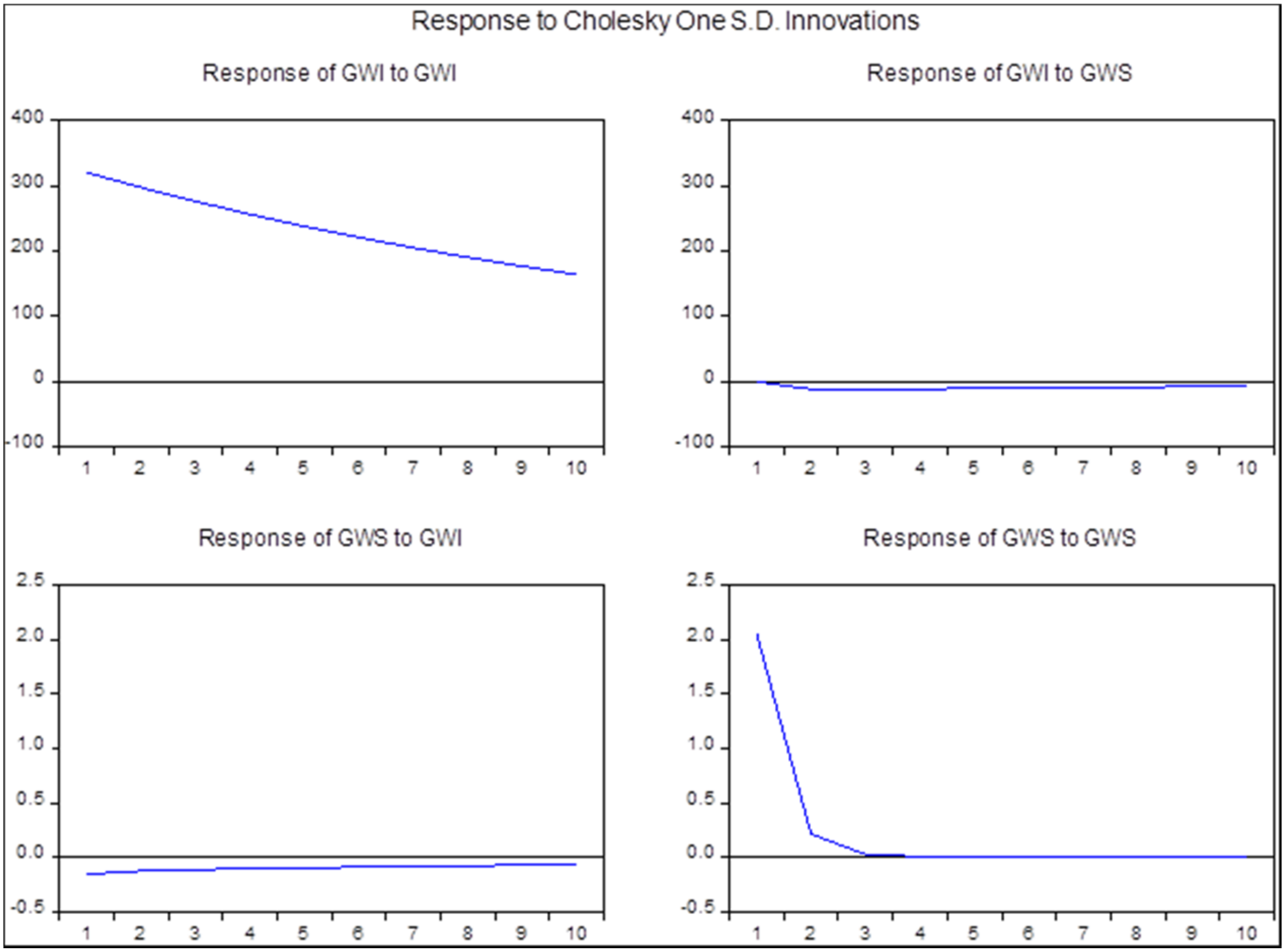

Fig. S4: Impulse response functions of GWS and GWI in Gujarat. X-axis represents response time and Y-axis represents response, respectively

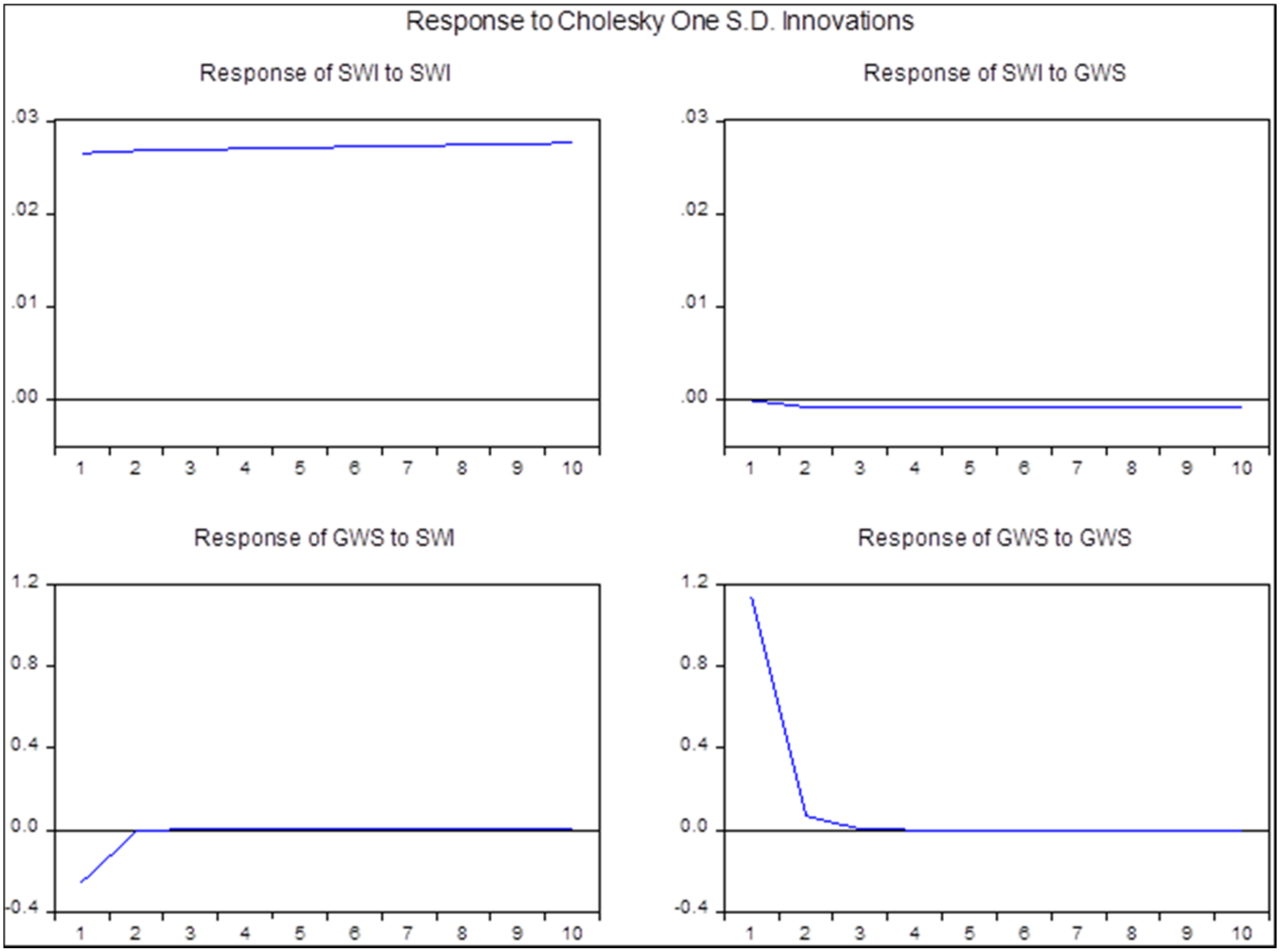

Fig. S5: Impulse response functions of GWS and GWI in Andhra Pradesh. X-axis represents response time and Y-axis represents response, respectively

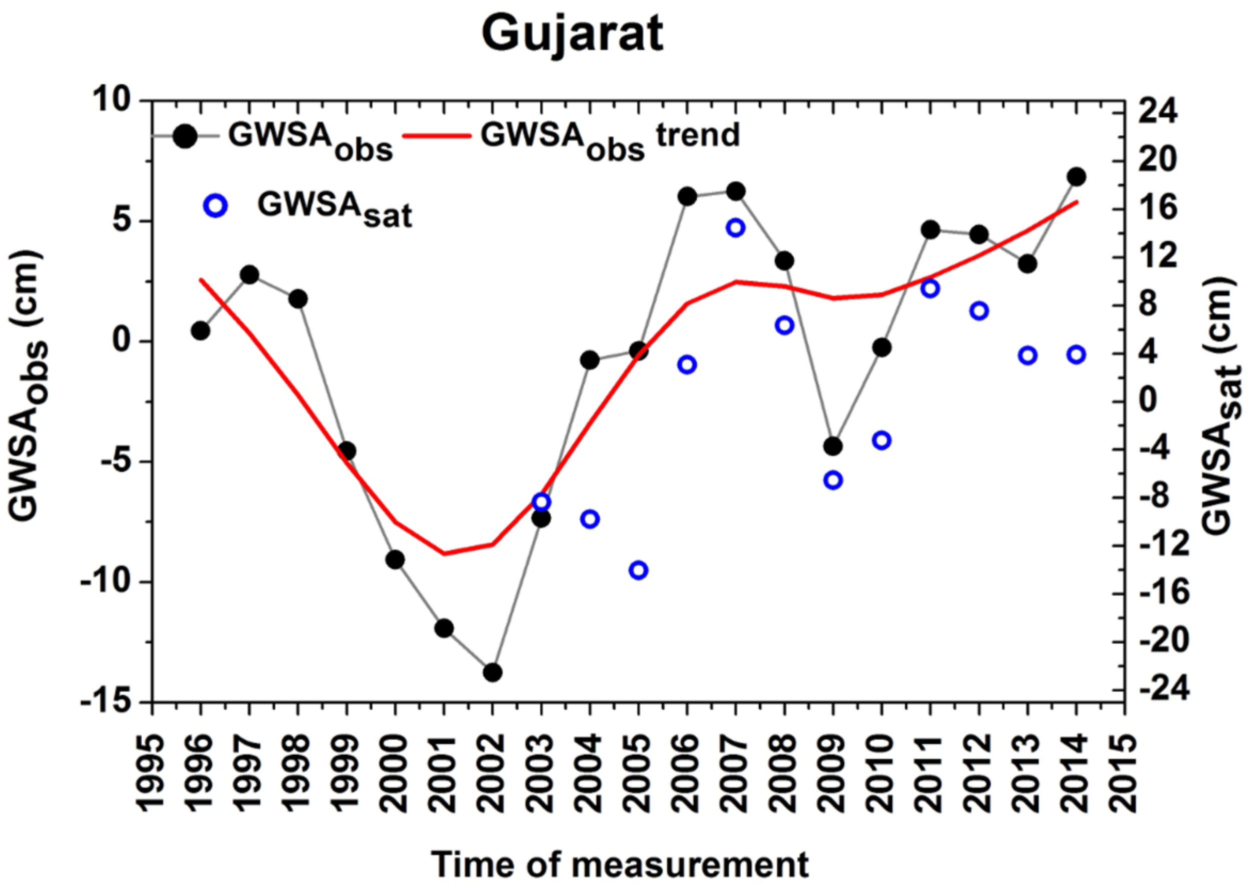

Fig. S6: Mean annual GWS_{obs} for Gujarat (n=177 locations). The data are fitted with HP trend (red line). Mean annual GWS_{sat} are shown using blue open circles

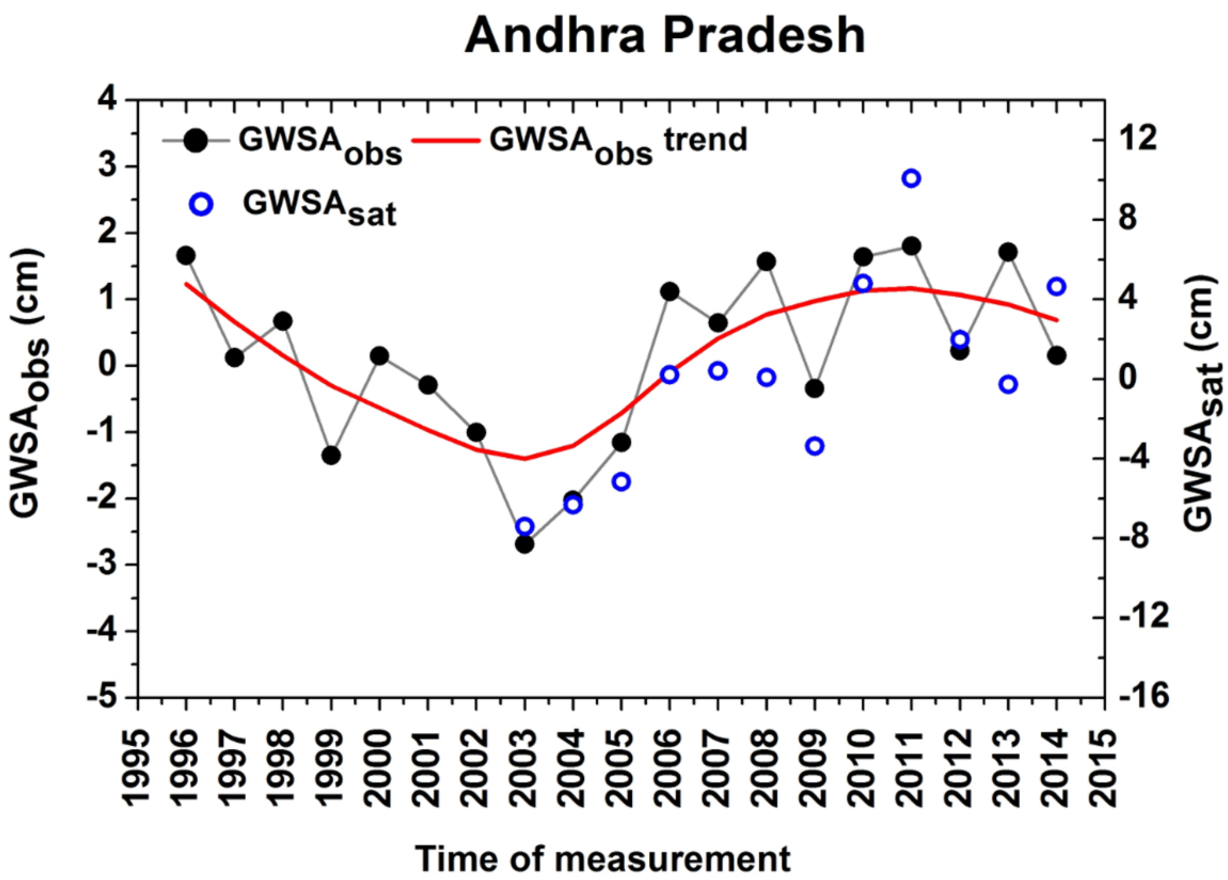

Fig. S7: Mean annual GWS$_{obs}$ for Andhra Pradesh (n=350 locations). The data are fitted with HP trend (red line). Mean annual GWS$_{sat}$ are shown using blue open circles

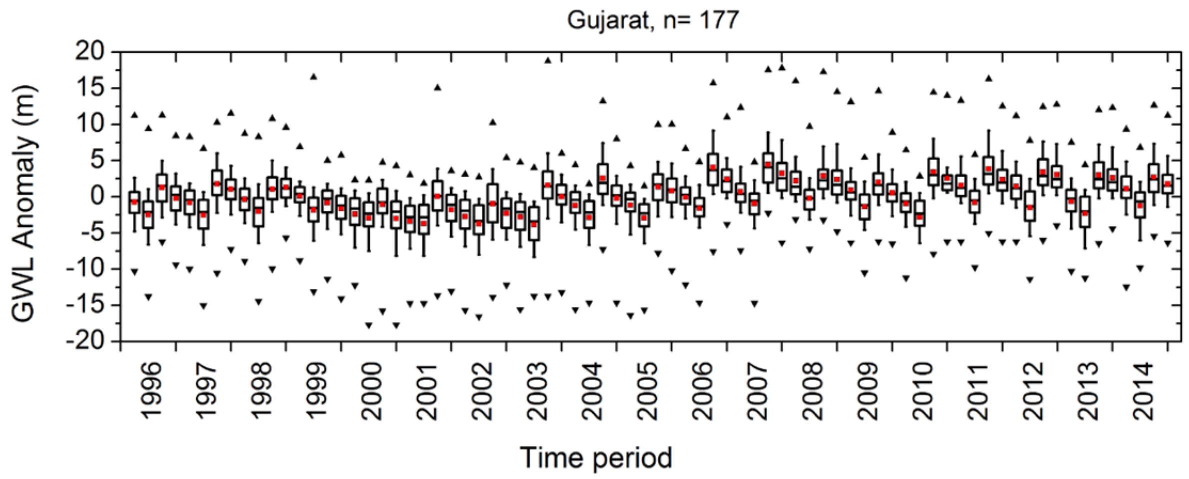

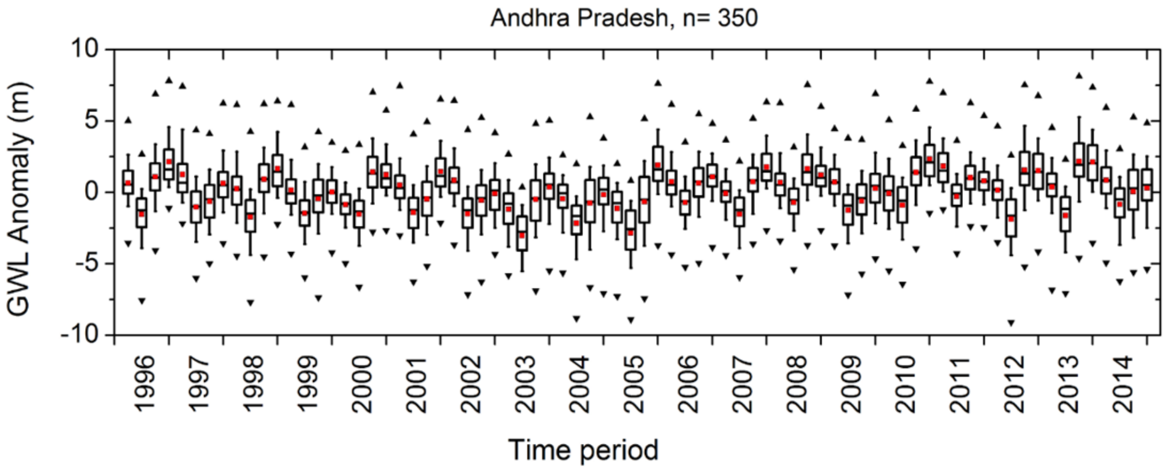

Fig. S8: Box-whisker plots of GWL anomaly for all the seasons for (a) Gujarat (GJ) and (b) Andhra Pradesh (AP) for 1996-2014. The extent of each box indicates the inter-quartile range (25 to 75th percentile) of the data; horizontal line within the box specify median values; red filled circles inside the box show mean values; upper and lower limits of whisker indicate $\pm 1\sigma$ deviation from the mean; top and down black filled triangles showing 99% and 1% data, respectively

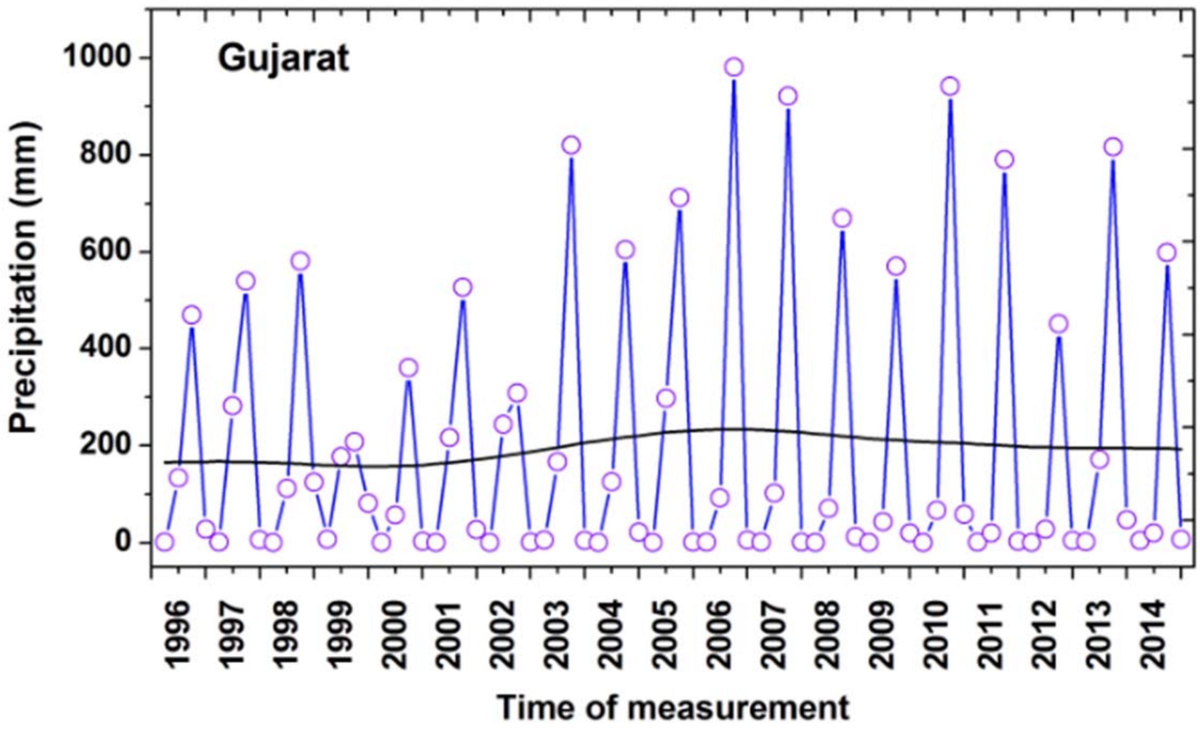

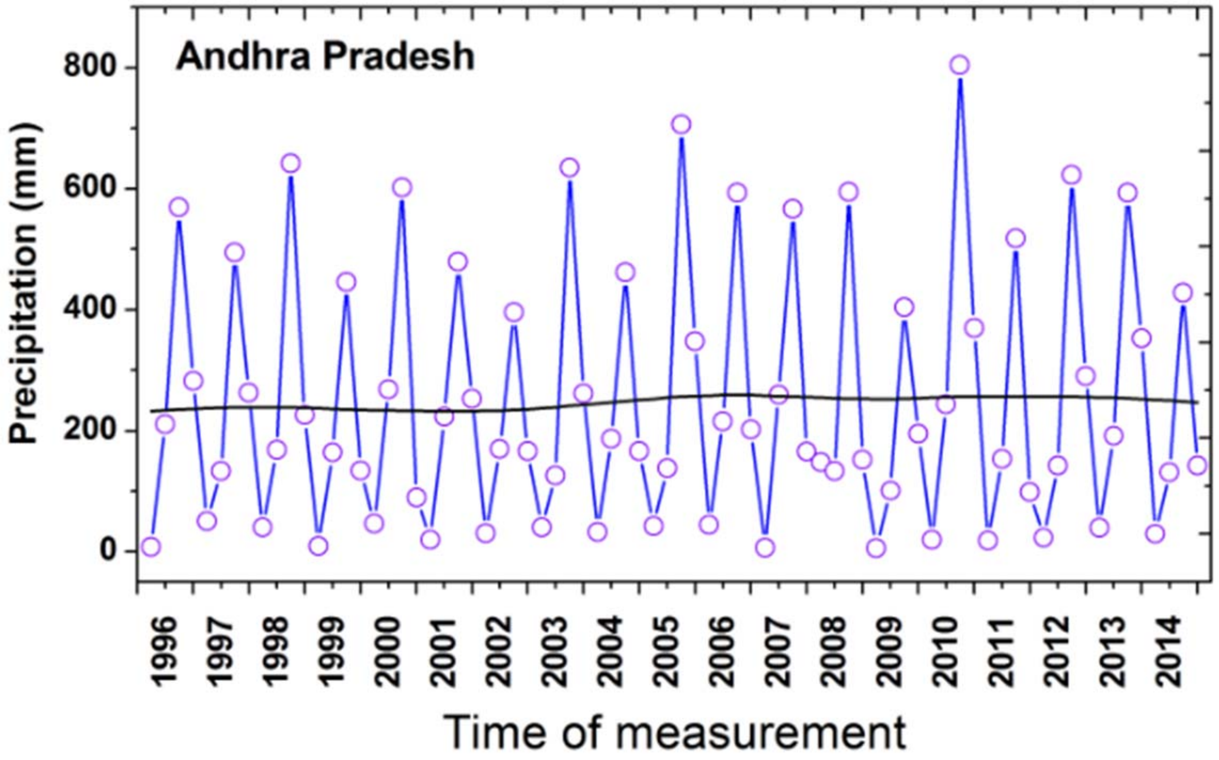

Fig. S9: Total seasonal precipitation and their HP trends for Gujarat and Andhra Pradesh

S6 Specific yield information

High-resolution specific yield map has been created using hydrogeology map of India (Fig. 1c). The map is constructed following CGWB (2012b) and Mukherjee et al. (2015). We segregate the S_y information from the CGWB database (CGWB, 2012) and assigned the data according to aquifer characteristics shown in Fig. 1c. The range in S_y values were determined from long-term pumping test data conducted over the years (MWR, 2009). On the basis of available information, we prepare a gridded ($0.1^0 \times 0.1^0$) S_y map for the Indian region. A unique specific yield value is assigned to each of the selected wells based on their geographic location. Observed GWS anomaly has been calculated by multiplying S_y and Δh values at all the in-situ well locations.
